# Supplementary material for: Scalable and rapid nearest neighbor particle search using adaptive disk sector
Source: PLoS One. 2025 Mar 20;20(3):e0311163. doi: 10.1371/journal.pone.0311163 (PMC11925300; doi:10.1371/journal.pone.0311163)
Supplement: Attachment — Submitted filename: Response_to_Reviewers.docx [file pone.0311163.s001.docx]

**Reviewer#1, Concern #1:** The contribution should be listed correctly. The context of the contribution is vague.?

**Author response:** The technical contributions of the method proposed in this paper, and its potential applications, are as follows:

- Technical contribution

1. We introduce a method that can rapidly define the search range using a disk sector-based approach based solely on particle velocity, without the need for explicit data structures like commonly used spatial data structures such as hash tables or K-d trees. While calculating anisotropic features typically requires computationally intensive processes like Singular Value Decomposition (SVD), in this study, we were able to quickly define the search range using only the particle's velocity and angle.
2. We propose a method for calculating the search range for moving particles by introducing a kernel that can reliably update the disk sector based on particle velocity.

- Potential applications with our method

1. Based on our method, we can rapidly construct NNPs in the direction of particle velocity and extend this approach to compute SPH simulations using an ellipsoidal kernel.
2. We can extend the algorithm to rapidly construct NPPs in the direction of particle velocity and then determine the conditions under which diffuse particles should be generated and initiate their creation.
3. Our algorithm can be extended to construct NNPs in the direction of velocity, compute a particle-based level set, and then extract isolines using the marching cubes algorithm.
4. Strands such as hair or fur are composed of particles, and our algorithm can be extended to handle self-collision by constructing NNPs using the method proposed in this paper to identify candidate particles for collision handling.
5. Our algorithm can be extended to calculate turbulent flow in a particle-based system by constructing NNPs based on particle velocity, allowing for the generation and maintenance of turbulence.

**Author action:** We have modified the above information (Line 89 in revised manuscript).

**Reviewer#1, Concern #2:** Any justification of why Equation (1) must be considered in this case?

**Author response:** First of all, thank you for your valuable feedback. In this study, we focused on how to efficiently generate and update disk sectors in response to particle movements. Although many existing methods model shapes like disks or cones, we chose to use a relatively simple implicit function form to perform updates efficiently. To initially filter particles with minimal computation, we utilized a sphere equation. As far as we know, our paper is the first to propose a method for generating and updating disk sectors to construct NNPs. While there are still many challenges to address, we plan to further optimize and improve this algorithm in the future.

**Author action:** We have modified the above information (Line 838 in revised manuscript).

**Reviewer#1, Concern #3:** Kindly justify the convergence of Equation (7) in relation to your proposed work.

**Author response:** In this paper, we use a weight w to automatically adjust the search range based on the velocity of moving particles. A parameter is required to define the search range per angle, and we interpolated the angle based on user-defined minimum and maximum values. However, if the velocity is too fast or too slow, the search range may excessively expand or contract, making it difficult to stably calculate NNPs. To address this issue, we introduced $w^{*}$, a refined weight based on a kernel, to determine the angle-based search range. As shown in Fig. 4, $w^{*}$ converges to a kernel value of 1, preventing it from growing uncontrollably and allowing us to calculate NNPs more stably.

**Author action:** We have modified the above information (Line 340 in revised manuscript).

**Reviewer#1, Concern #4:** As for Fig 5, the author uses brute force which is the worst way to find the optimal solution. Any justification on this?

**Author response:** Thank you for your insight. In fact, this study did not only experiment with the brute force method; we also experimented with various approaches such as hash tables and *K*-d trees, tailored to different scenarios. Fig 5 illustrates the brute force method, which is the simplest way to demonstrate NNP without considering moving particles, but our method is not limited to this approach.

**Author action:** Thanks for the good comment.

**Reviewer#1, Concern #5:** Fig 11 should be explained?

**Author response:** Fig. 11 shows the computation time for the scene used in Fig. 10, experimented with hash tables and K-d trees, represented with rainbow colors. Both the hash table and K-d tree methods show blue colors when the number of particles is small, indicating that NNP can be computed quickly. However, when the number of particles is large, the search time increases for both hash tables and K-d trees, resulting in relatively longer computation times. Particularly at boundary regions, hash tables exhibit more pronounced aliasing effects compared to K-d trees. This occurs because the K-d tree supports multi-resolution as a spatial data structure, leading to smoother color transitions even near boundaries with significant density differences. In contrast, the hash table displays greater variations in search time near these boundaries due to density differences, which is visualized as aliasing. The time differences within dense regions for the K-d tree are primarily due to differences in search performance depending on tree depth, whereas the hash table, which has relatively constant-time complexity, shows less variation in computation time.

**Author action:** We have modified the above information (Line 463 in revised manuscript).

**Reviewer#1, Concern #6:** As for Table 2, please conduct Friedman test to validate the superiroty of your proposed work. You may refer/cite the following work

(a) Supervised learning perspective in logic mining

(b) Novel logic mining incorporating log linear approach

Please conduct sufficient hypothesis testing to validate your work

**Author response:** We conducted experiments on the results of this study using the Friedman test as you suggested, and the relevant findings have been added to the main text.

**Author action:** We have modified the above information (Line 542 in revised manuscript).

**Reviewer#1, Concern #7:** The author should conduct convergence analysis to validate the proposed work. As for application, please justify how do you choose the applications. Any citation to support your justifcation?

**Author response:** Thank you for your valuable comments. The method proposed in this paper can be applied to techniques in Computer Graphics that require NNP. Most methods for calculating NNP analyze their performance by measuring computation time. Similarly, our method was compared and analyzed across various scenarios, not just a single static scenario, and was further validated by applying it to different applications to demonstrate its versatility. Frankly, this is the first time I’ve encountered convergence analysis being mentioned in the context of NNP techniques within the Computer Graphics field. However, I believe it could have significant value. If the Friedman test you mentioned falls under this category, I will use it to analyze our proposed method. While this may not be a commonly used analysis technique in our main field of Computer Graphics, I will conduct experiments to analyze the content as suggested.

**Author action:** Thanks for the good comment.

**Reviewer#1, Concern #8:** Qualitative analysis should be conducted in all applications?

**Author response:** Thank you for the insightful question. The method proposed in this paper focuses on efficiently calculating NNP, and the best way to demonstrate its performance is by measuring the trade-off between quality and efficiency. The ellipsoidal-kernel-based particle-based fluids and diffuse particles, shown in various applications, are modeled based on the momentum of neighboring particles. While they appear similar in terms of visual quality, our method shows improved efficiency by finding NNPs more effectively. This is demonstrated across various scenarios in the Results section. The diffuse particles and surface tracking sections reveal visually well-represented outcomes. Additionally, collision detection was performed effectively without any missed collisions. Since our approach enhances computational performance by reducing the number of candidate particles when calculating NNPs, it shows no significant difference in terms of quality. Nevertheless, even in the final application, turbulent flow, we observed that turbulence was generated and maintained well, with no noticeable noise compared to previous methods (see Fig. 23).

**Author action:** We have modified the above information (Line 812 in revised manuscript).

**Reviewer#1, Concern #9:** The conclusion must be mapped with the contributions?

**Author response:** We have corrected.

**Author action:** We have modified the above information (Line 779 in revised manuscript).

**Reviewer#2, Concern #1:** The introduction is quite generic, please detail in a wider way to extend their main contribution to other research fields . There is no in-depth discussion about the advantages of the technique proposed in comparison between state of the art . Please add citations for this section.

**Author response:** Thank you for the valuable advice. In the Introduction section, we have separated and explained the technical contributions and the potential applications of the proposed method. Additionally, we have described various approaches that can be applied and included related research in the Related Work section.

**Author action:** We have modified the above information (Line 83 and 229 in revised manuscript).

**Reviewer#2, Concern #2:** Expression a¿b=c is unclear Line 490.

**Author response:** That was a typo, and I have corrected it as follows : $a>b=c$.

**Author action:** We have modified the above information (Line 560 in revised manuscript).

**Reviewer#2, Concern #3:** Cases for Fig 2. (A), (B), and (C) , should use lowercase letters.

**Author response:** Fig. 2 consists of three subfigures, with subcaptions labeled as (a), (b), and (c). However, using lowercase letters within the subfigure captions would overlap with these labels, making them difficult to distinguish. Therefore, we have used uppercase letters for clarity, and we appreciate your understanding.

**Author action:** Thanks for the good comment.

**Reviewer#2, Concern #4:** use the symbol δ instead of delta to define weight in line 288 from Eq(4).

**Author response:** We have corrected.

**Author action:** We have modified the above information (Line 329 in revised manuscript).

**Reviewer#2, Concern #5:** Axis from Fig 4 needs to be detailed or described.

**Author response:** We have corrected ($X$-axis : $w$(see Equation 7), $Y$-axis : weight value $w^{*}$).

**Author action:** We have modified the Fig 4 caption.

**Reviewer#2, Concern #6:** Fig 5a and Fig 5b are not explained.

**Author response:** We have corrected. “Fig. 5a and Fig. 5b show the results of calculating neighboring particles based on the moving target particle over time.”

**Author action:** We have modified the above information (Line 381 in revised manuscript).

**Reviewer#2, Concern #7:** From Fig 9 (X-axis : frame #, Y-axis : number of NNPs) I recommend put these in the corresponding axis of each Figures.

**Author response:** Thank you for the good suggestion. We also believe that including axis information in Fig. 9 would be beneficial. However, due to the small size, it may not be clearly visible. Therefore, we have provided the information in the caption.

**Author action:** Thanks for the good comment.

**Reviewer#2, Concern #8:** There are K-d trees in the manuscript that are not italicized.

**Author response:** We have corrected.

**Author action:** Thanks for the good comment.

**Reviewer#2, Concern #9:** It would be gratefully appreciated if the author can share the pseudocode and data set from the experimental set.

**Author response:** Thank you for the great suggestion. We plan to add the test code and related data to GitHub once the submitted paper is accepted.

**Author action:** Thanks for the good comment.

**Reviewer#3, Concern #1:** The paper is well written and clearly illustrated. Scalability could be a problem. It looks like the experiments have been done only on 10,000 particles and there are too many assumptions about the configuration of the particle at the point of collision. This can reduce the practicality of the solution.

**Author response:** Thank you for the insightful question. In this paper, we conducted experiments not only with 10,000 particles but also with various density maps composed of a larger number of particles (see Fig. 7). We also conducted experiments with 500,000 particles (see Fig. 13, Fig. 16b). Additionally, to test moving particles represented by simulations, we applied our method to ellipsoidal kernels, diffuse particles, and isoline tracking in particle-based fluids, confirming its efficiency. Furthermore, we applied our method to particle-based hair simulation and turbulent flow, yielding meaningful results. These findings demonstrate that the proposed method can be effectively utilized in a variety of applications involving a large number of particles.

**Author action:** Thanks for the good comment.
